# Association between Chromosome 4 and mercury accumulation in muscle of the three‐spined stickleback (*Gasterosteus aculeatus*)

**DOI:** 10.1111/eva.13298

**Published:** 2021-10-09

**Authors:** Federico C. F. Calboli, Vyshal Delahaut, Io Deflem, Pascal I. Hablützel, Bart Hellemans, Anna Kordas, Joost A. M. Raeymaekers, Lieven Bervoets, Gudrun De Boeck, Filip A. M. Volckaert

**Affiliations:** ^1^ Laboratory of Biodiversity and Evolutionary Genomics KU Leuven Leuven Belgium; ^2^ Department of Biology Systemic Physiological and Ecotoxicological Research (SPHERE) University of Antwerp Antwerpen Belgium; ^3^ Flanders Marine Institute (VLIZ), InnovOcean Site Oostende Belgium; ^4^ Faculty of Biosciences and Aquaculture Nord University Bodø Norway; ^5^ Present address: Natural Resources Institute Finland (Luke) Helsinki Finland

**Keywords:** adaptation, environment, mercury, next generation sequencing, pollution, selection, three‐spined stickleback

## Abstract

Anthropogenic stressors, such as pollutants, act as selective factors that can leave measurable changes in allele frequencies in the genome. Metals are of particular concern among pollutants, because of interference with vital biological pathways. We use the three‐spined stickleback as a model for adaptation to mercury pollution in natural populations. We collected sticklebacks from 21 locations in Flanders (Belgium), measured the accumulated levels of mercury in the skeletal muscle tissue, and genotyped the fish by sequencing (GBS). The spread of muscle mercury content across locations was considerable, ranging from 21.5 to 327 ng/g dry weight (DW). We then conducted a genome‐wide association study (GWAS) between 28,450 single nucleotide polymorphisms (SNPs) and the accumulated levels of mercury, using different approaches. Based on a linear mixed model analysis, the GWAS yielded multiple hits with a single top hit on Chromosome 4, with eight more SNPs suggestive of association. A second approach, a latent factor mixed model analysis, highlighted one single SNP on Chromosome 11. Finally, an outlier test identified one additional SNP on Chromosome 4 that appeared under selection. Out of all ten SNPs we identified as associated with mercury in muscle, three SNPs all located on Chromosome 4 and positioned within a 2.5 kb distance of an annotated gene. Based on these results and the genome coverage of our SNPs, we conclude that the selective effect of mercury pollution in Flanders causes a significant association with at least one locus on Chromosome 4 in three‐spined stickleback.

## 1 INTRODUCTION

It is widely accepted that planet Earth is experiencing a period of dramatic biodiversity loss (Ceballos et al., 2015; Dirzo et al., 2014; Johnson et al., 2017). Human activities can drive biodiversity loss even without direct targeting and exploitation of specific species, but simply by changing the natural environment, as in the case of habitat fragmentation, or chemical pollution (Mazor et al., 2018; Newbold et al., 2015). When it proves impossible for species to successfully adapt, populations and sometimes whole communities are extirpated (Brook et al., 2008; Dias et al., 2017; Wan et al., 2019). Yet in those cases where the effects of these stressors are sublethal, they create a selective pressure for adaptation (Laikre et al., 2010; Palumbi, 2001; Whitehead et al., 2012). In aquatic environments, metals are probably among the best known and most widely spread group of anthropogenic stressors that have the potential to induce sublethal effects to biota (Tchounwou et al., 2012). One example is mercury, which has received increased attention in the last decades for its neurotoxic effects towards humans and wildlife, tragically exemplified by two mass pollution events, in the Minamata Bay (Japan) and Bagdad (Iraq) (Amin‐Zaki et al., 1977; Kurland et al., 1960). As an ubiquitous element of the earth crust, mercury is naturally released into the environment as a consequence of volcanic eruptions and rock weathering. However, human activities such as mining, cement production, chloralkali plants, and metallurgy have been increasing the release of mercury to such an extent that international action for mitigation became indispensable. It has led to the drafting and signing of the Minamata Convention in 2017 (Amos et al., 2013; Outridge et al., 2018), yet leaving thousands of tonnes of historical mercury emissions still in the environment (AMAP/UN Environment, 2019). Inorganic mercury may enter surface waters via direct discharges or atmospheric deposition, where, in anoxic settings with a substantial fraction of bioavailable mercury species, it can be converted to the organometal methylmercury (MeHg) by bacteria (Hsu‐Kim et al., 2013). In its organic form mercury is highly persistent in biological tissues resulting in strong biomagnification of MeHg along the food chain. Consequently, species taking up moderate or high positions in the food web, such as predatory fish and birds, are exposed to higher levels of MeHg compared to species at lower trophic levels (Wiener et al., 2002). High levels of mercury exposure are known to lead to elevated accumulation in crucial organs such as gonads, muscle and nervous tissue, where it can exert toxic effects which alter the overall fitness of the organism (Pereira et al., 2019).

Although the toxicokinetics of acute mercury exposure have been well explored, the potential role of mercury as a selective agent driving contemporary evolution is still to be investigated. The finding by Ruuskanen et al. (2020), that the rise in mercury emissions coincided with an increased selection for mercury detoxification in bacterial communities, does suggest a selective effect of mercury on biota. In another study, it was shown that Artemia populations from historically polluted mining regions portrayed higher survival at high mercury concentrations than strains that were recently introduced (Pais‐Costa et al., 2020). However, the mechanisitic nature of this increased tolerance is yet to be determined and little is known about the genes involved in adaptation to mercury pollution, which is one of the drivers of our work. Studies on other heavy metals and organic compounds provide evidence for evolutionary responses in higher organisms, due to either *de novo* mutations or acting on standing genetic variation (Oziolor et al., 2017). To date, the best‐substantiated evidence is provided by the Atlantic killifish (*Fundulus heteroclitus*) system, where targeted selection at the aryl hydrocarbon receptor‐based signalling pathway has mediated adaptation to urban pollution (Reid et al., 2017).

Here, we used the three‐spined stickleback *Gasterosteus aculeatus* Linnaeus, 1758 (Gasterosteidae), as a model for adaptation to mercury pollution in natural populations of freshwater ecosystems. The three‐spined stickleback is increasingly used as a sentinel species in ecotoxicological studies (Catteau et al., 2020; Sanchez et al., 2008; Webster et al., 2017), and is a well‐established eco‐evolutionary model (El‐Sabaawi, 2017; Hendry et al., 2013; McKinnon & Rundle, 2002). This species has shown genetic adaptation ranging from single genes of large effect (Chan et al., 2010; Colosimo et al., 2005) to multiple genes of small effect (Jones et al., 2012). The region of this study is the Flemish (Belgium) riverscape, where sticklebacks dominate the pollution‐stressed fish communities of aquatic habitats such as ditches, channels, ponds and streams (Bervoets et al., 2005; Breine & Van Thuyne, 2015; Raeymaekers et al., 2017). This region has experienced a significant degree of metal pollution from industrial activities since the 19th century, with very local though scattered signatures of mercury contamination in soils and sediments (Tack et al., 2005; Van Steertegem, 2011).

We therefore hypothesize that nonmigratory stickleback populations in Flanders have experienced strong, mercury‐induced selection at some locations. To test this assumption, we first assessed the current pollution status by quantifying the mercury levels in the sediment and the levels in the fish at 21 locations across three basins. We then investigated the genetic basis of adaptation to mercury pollution and therefore conducted a genome‐wide association study (GWAS) between SNPs (single nucleotide polymorphism) and the mercury levels in the fish as the phenotype. In case pollution‐induced selection has occurred (Bickham, 2011), we would be able to find a significant association between genotype and phenotype (hypothesis 1). Assuming that hypothesis 1 is correct, we use the results of our analyses to test whether mercury exposure has led to parallel adaptation to mercury tolerance based on different loci in different populations, or whether the same SNP(s) is (are) associated with mercury associated phenotypes in all populations in a consistent way (hypothesis 2). We discuss our results in the light of these hypotheses and in the context of the selective effects that historical mercury pollution may have caused to fish populations.

## 2 MATERIALS AND METHODS

### 2.1 Sampling and sample preparation

**FIGURE 1:**
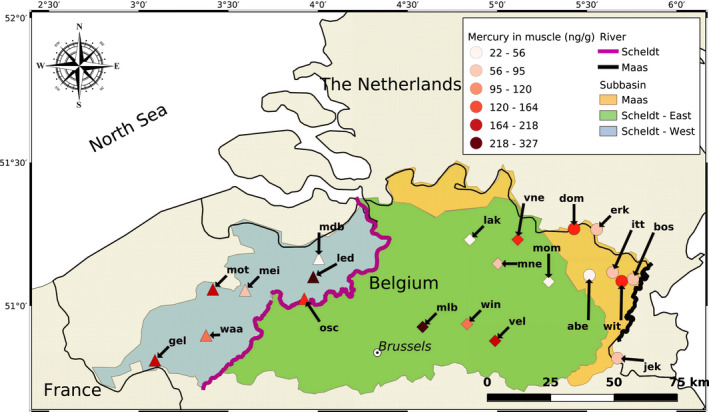
Map of the sampling region of three‐spined stickleback. In yellow, the Flemish part of the drainage basin of the Maas; in green, the drainage basin of the Scheldt‐E; in blue, the drainage basin of the Scheldt‐W. Each sampling location is identified by a unique three letter code based on the name of the location (Table S1). The colour of each sampling location represents the range of the amount of mercury in muscle of the fish sampled at that location; the symbol indicates the basin each sample belongs to (Scheldt‐West: triangle; Scheldt‐East: diamond; Maas: circle)

We sampled resident three‐spined sticklebacks from 21 locations across three drainage basins (seven locations per basin) in Flanders (Belgium), namely river Maas, eastern basin of the Scheldt river (Scheldt‐E), and western basin of the Scheldt river (Scheldt‐W) with permission of the Agency Nature and Forest (ANB) (Figure 1 and Table S1). Locations were chosen based on information from the Research Institute Nature and Forest (INBO) on the mercury content in European eel *Anguilla anguilla* (Bonnineau et al., 2016), the Flemish Environmental Agency (VMM) on the mercury content in sediment and presence of three‐spined stickleback from the fish databank (www.vis.inbo (Brosens et al., 2015)). At each location, 25 fish were collected with dip nets (with exception of the Itterbeek, where only 23 fish could be collected). Fish were then transferred to an oxygenated bucket filled with 25 L of water collected on site and transported to the University of Antwerp (Antwerp, Belgium). In addition, five replicate sediment samples were taken with a petite Ponar grab sampler, filtered over a 1‐mm sieve to remove bigger debris and stored in 50‐ml Falcon tubes. The day following collection, fish were euthanized using an overdose of buffered Tricaine mesylate (MS‐222) according to approval for the project of the Ethical Commission Animal Experiments of the KU Leuven. Length and weight of each fish were individually measured. Fish were dissected on a glass plate kept on ice to maximize tissue preservation. In those cases where gonads were mature, we recorded the sex of the sample. For each fish, the caudal fin and tail peduncle were removed and stored in ethanol for DNA extraction. For mercury analysis, muscle tissue on both flanks was dissected from the posterior end of the dorsal fin (spines) to the caudal peduncle (75 mg of tissue on average). The skin was removed and discarded, whereas the muscle tissue was snap‐frozen in liquid nitrogen and temporarily stored at −80℃.

### 2.2 Mercury analysis in muscle tissue and sediment

The muscle tissues and sediment samples were freeze‐dried for 48 h and acid digested according to Verhaert et al. (2019). Metal analysis of the samples was performed using a high‐resolution inductively coupled plasma‐mass spectrometer (HR‐ICP‐MS, Thermo Scientific). Certified freeze‐dried mussel tissue (BCR 2976, NIST, USA), channel sediment (BCR‐320R, IRMM, Geel Belgium) and procedural blanks were included for quality control (Table S2). The total (inorganic and organic) mercury content in muscle tissue is further reported in ng/g dry weight (DW), and total mercury in sediment as µg/kg dry solids (DS).

### 2.3 Genotyping

DNA was extracted from the tail peduncle of each fish following a standard protocol (Cruz et al., 2017). Briefly, each sample underwent Proteinase K (Sigma) digestion in SSTNE buffer, followed by RNA removal with an RNAase cocktail (Thermo Fisher Scientific), salt purification, isopropanol DNA precipitation and ethanol DNA washing. DNA concentration was then measured using Quant‐iT Picogreen dsDNA assay kit (Thermo Fisher Scientific, Waltham, USA). For each sample, 10 µl of genomic DNA was standardized at a concentration of 10 ng/µL and digested at 75°C using the restriction enzyme *ApeKI* (New England Biolabs). After digestion, Illumina adapters (with barcodes) were ligated to the fragments, to allow pooled sequencing. The purified library DNA was then amplified with 18 PCR cycles. After PCR, 20 ng of 96 samples were pooled and sent for DNA sequencing to the Genomics Core at the KU Leuven (Leuven, Belgium). Fragments of size 240 to 340 bp (base pairs) were selected with a BluePippin unit (Sage Science) and sequenced on an Illumina HiSeq 4000 (San Diego, USA) platform to produce paired‐end reads of 150 bp. The 523 samples were divided into six 96‐sample libraries, with the final sixth library composed of samples that had not yet been genotyped and samples whose genotyping had failed in the first five libraries. This approach allowed us to minimize the number of samples discarded from the analysis due to poor genotyping. The results from the Illumina genotyping were then demultiplexed using the ‘process_radtags’ module of STACKS v1.28 (Catchen et al., 2013), after which the genomic data of each sample were aligned to the stickleback reference genome produced by Jones et al. (2012) using Bowtie 2 v2.3 (Langmead & Salzberg, 2012); we retained fragments that had an unique match to the genome. Individual genotypes were then called using FreeBayes v1.3.1‐1 (Garrison & Marth, 2012). As the accepted standard for SNPs, we removed all SNPs that were not biallelic, and we also removed any SNP with a failed genotyping rate greater than 10%, and all sampled individuals with more than 25% of their genome data missing, bringing our final dataset to 28,450 SNPs and 512 individuals (a coverage of approximately one SNP for every 25 kb). In all analyses, we used 507 samples due to the failure of the mercury assessment in five samples. We did neither test nor correct for Hardy–Weinberg equilibrium because we assumed that mercury exerts a selective effect on the fish, and thus removing SNPs based on Hardy–Weinberg equilibrium would effectively remove all possible signals from the data. Because we used unique alignments to the reference genome, we do not envision paralogs would affect marker HWE.

### 2.4 Data analysis

#### 2.4.1 Population structure

To test the population structuring in our samples, we followed the analyses in Calboli et al. (2010) and Astle and Balding (2009). Starting from genotype data coded as 0/1/2 (to indicate the number of rare alleles in each genotype, with 0 being homozygous for the common allele, 1 being heterozygous and 2 being homozygous for the rare allele), we first used a *z*‐score transformation to normalize and center the data for each SNP. We then replaced all missing genotypes with the mean z‐score of that SNP (this procedure was carried out for all SNPs). After transformation and removal of missing data, the resulting genetic matrix is the matrix *X*. We computed the kinship matrix *K* as *K* = XX’/2*n*, where *X*’ is the transposed of the *X* matrix, and 2*n* is twice the number of SNPs in the *X* matrix. The eigenvectors of the kinship matrix represent the principal components of the matrix and can be used to test for population structure. The kinship matrix was also used to calculate the pairwise *F*_st_ between each population, according to the Ochoa and Storey approach (Ochoa & Storey, 2021) implemented in the R package popkin 1.3.6.

#### 2.4.2 Sex determination

We used a simple logistic model to associate the sex determination we obtained by observing the gonads at dissection and genotype in base R, as:

sex ~ genotype

#### 2.4.3 Analysis of mercury in muscle

Because we assume that the amount of mercury that accumulates in muscle is dependent on the amount of mercury in the environment and exposure time (age), our preliminary analysis was to conduct a linear model on mercury in muscle over fish length (as a proxy for age) and a second linear model where mercury in the sediment was added as second term to the first model. After taking the square root of the mercury value to respect the assumption of normality, the models were run in base R as:

model 1 = sqrt(mercury) ~ location:length
model 2 = sqrt(mercury) ~ location:length +mercury in sediment
anova(model 1, model 2)

to test the effect of fish length within each location, since we cannot assume that growth rates and fish ages match across locations, and to test whether adding mercury in sediment would significantly increase the amount of variance explained by this term in the model.

To investigate the difference in both the mercury accumulation in the muscle tissue of the fish populations across the three basins, as well as the mercury contamination in the sediment across basins, an analysis of variance (ANOVA) was run on these two models:

sqrt(mercury in muscle) ~ basin +location:length + (1|location)
sqrt(mercury in sediment) ~ basin

Differences between basins were analysed using orthogonal contrast between the levels of ‘basin’. The first contrast was between the effect of the Maas basin vs. the mean effect of the two Schelde basins, and the second contrast was between the SW and SE, ignoring the Maas.

#### 2.4.4 Genome‐wide association study

We expect two main factors affecting the assumption of independence of errors in the samples: the effects of population (the samples show higher kinship within population rather than between), and the effects of shared environment. An ulterior difficulty is caused by the fact that both these effects act at the same time in each sampling location, in a way that does not allow to separate the effects of kinship and the effects of shared environment. Therefore, for each SNP, we employed a linear random effect model to test for association between mercury in muscle as a dependent variable, and genotype as the main independent variable. The reason is that we explicitly parametrize sampling location as a random variable to correct for the nonindependence of errors within location. This approach avoided making subjective decisions on what genetic and environmental variables to choose as random factors. Other fixed effect independent variables were sex and length estimated within sampling location. Body size is highly associated with mercury in muscle, but we assume that due to ecological differences growth rates differ between locations. Hence, the estimation of the effect of length within each sampling location proves more correct than assuming that fish of the same length are of comparable age if they come from different locations. Before running the model we normalized the dependent variable—mercury in muscle—by square root transformation. In summary, the linear mixed model was overall parametrized as:

sqrt(mercury in muscle) ~ SNP genotype +sex + location: length + (1|location)

In order to directly assess the effect size of the SNP showing the strongest association with mercury, we z‐score transformed the mercury data and used the very same mixed model used for the association analysis for this SNP alone. This approach provided a standardized beta coefficient of the regression for the SNP, which is a measure of the effect size on the variance of the dependent variable for every allele change. All linear models were run in R 4.0.3, and the mixed model analyses were done using the R package LME4 1.1–26 (Bates et al., 2015). In a second approach, we used a latent factor mixed model (LFMM) analysis to test for association between genotype and muscle heavy metal content (implemented in the R package lfmm 1.0 (Frichot et al., 2013). Following Frichot, and based on the amount of variance explained, the first seven principal components were selected as the main components of the variance in the data. A LFMM analysis takes the genotype matrix as the dependent variable; hence, we chose mercury in muscle, mercury in sediment (to represent environmental variability across locations), sex and length as independent variables. The parameters of the analysis were estimated through ridge regression, and the *p*‐values of the GWAS were calibrated using a genomic inflation factor.

#### 2.4.5 *F*_st_ outlier test

We also tested whether populations in a highly polluted environment showed signs of selection at the genetic level over and above any population structuring. We assessed the presence/absence of differentially selected loci using the outlier test approach used in OutFLANK (Whitlock & Lotterhos, 2015). To avoid any false positive caused by sex effects, we did not include Chromosome 19 in the outlier analysis. Explicitly grouping the samples based on the 21 sampling location circumvents any subjectivity in how to otherwise group the samples, and tests whether there are loci that are under selection across all populations at once. To directly test our second hypothesis, that of the selection of parallel genetic basis of adaptation, we also divided the samples by river basin (Maas, Scheldt‐W, Scheldt‐E) to assess whether we have evidence of loci that are differentially selected across basins.

#### 2.4.6 Gene ontology analysis

We investigated the most significant GWAS and OutFLANK hits in detail by screening a genomic sequence of 2500 bp up‐ and downstream of the SNP for the presence of annotated genes. For SNPs exactly located on an mRNA coding region (exon) of a gene, we aligned the three potential amino acid (AA) sequences given by Ensembl to the AA sequence reported in the UniProt database (https://www.uniprot.org) to verify the SNP position in the codon and determine the effect of the mutation on the amino acid sequence. We also used the Biomart tool from Ensembl (https://www.ensembl.org/biomart) to query the list of annotated genes on chromosomal regions identified in the analysis. We co‐extracted gene names, ontology terms and position, and subsequently selected the genes with GO term names that imply involvement in cellular metal binding processes. For the latter criterion we filtered those genes with the GO term name ‘metal ion binding’ and ‘zinc ion binding’. Finally, the level of significance of the GWAS SNPs that fell within these genetic regions was identified to assess the probability that they play a role in mercury accumulation.

## 3 RESULTS

### 3.1 Population structure and sex determination

**FIGURE 2:**
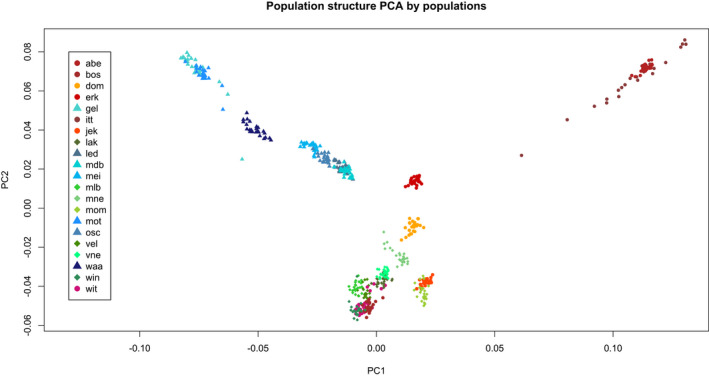
PCA plot (PC1 and PC2) of the population structure of all samples, after removing SNPs on Chromosome 19 (the sex chromosome). Populations represented with triangles of a shade of blue have been sampled in the Scheldt‐W basin; populations represented with diamonds of a shade of green have been sampled in the Scheldt‐E basin; populations represented by circles of a shade of brown have been sampled in the Maas basin

Plotting the first two components of the PCA revealed that the population structure of the 21 sampling locations matches the division of the sampling area in three drainage basins (Figure 2), with some overlap between the Maas and Scheldt‐E drainage basins. This observation confirms an earlier analysis based on microsatellites (Raeymaekers et al., 2012) and implies that the divide between the Maas and Scheldt‐E basin has not been stable between the Last Glacial Period and today. We observed that SNPs on Chromosome 19 are strongly associated with phenotypic sex (median ‐log_10_(*p*‐value) = 38; Figure S1), an expected result because Chromosome 19 is the sex chromosome (Peichel et al., 2004). Plotting the main components showed a peculiar pattern (Figure S2), with populations split in two groups. We interpreted this result as the effect of the sex chromosome on the clustering: the effect was especially evident when plotting PC3 and PC5 (Figure S3a,b). Once the analysis was run again removing Chromosome 19, the split of the populations in males/females disappeared (Figure 2). Because the sex chromosome causes a clustering pattern on PC3, we tested this clustering with a chi‐square test between the samples we sexed phenotypically during dissection, obtaining a *p*‐value (on one degree of freedom) lower than 2.2 × 10^−16^; thus we used the clustering on PC3 to assign a ‘genetic sex’ to all samples, including those we could not phenotypically sex. Genetic sex was then used in all subsequent analyses. The agreement of our genome‐wide SNP data with the broad population structure of three‐spined sticklebacks in Flanders (Raeymaekers et al., 2012) and with the chromosomal sex determination in three‐spined stickleback (Peichel et al., 2004) indicates that our SNP data are reliable and provides biologically meaningful results. Pairwise *F*_st_ analysis reveals that the populations in analysis are well diversified, with a mean pairwise *F*_st_ of 0.22 (range 0.08–0.38), indicating low admixture between them (Table S3).

### 3.2 Mercury pollution

**FIGURE 3:**
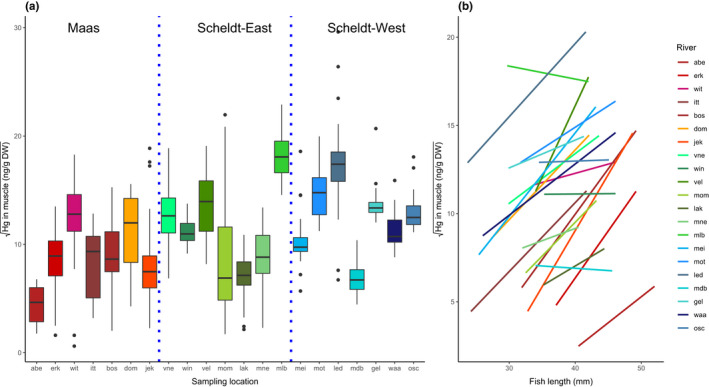
(a) Boxplot of the distribution of the total mercury in muscle [in ng Hg per gram dry weight (DW)]. In a shade of brown the populations sampled in the Maas basin; in a shade of green populations sampled in the Scheldt‐E basin; in a shade of blue populations sampled in the Scheldt‐W basin. (b) Regression lines of the square root of mercury in muscle over fish length for all the sampling locations—the colour scheme follows plot (a)

A considerable spread of muscle mercury content across stickleback populations was observed, with median levels ranging from 21.5 ng/g DW at the Abeek (Maas) to 327 ng/g DW at the Molenbeek (Scheldt‐E) (Figure 3a). There was a significant difference in mercury bioaccumulation between the three basins (*F*_2,504_ = 31.5, *p* < 0.001, all pairwise *post hoc* comparisons with Tukey Honest Significant Difference test <0.05), with the least polluted populations found within the Maas basin and significant higher concentrations measured in the fish of the Scheldt‐E and Scheldt‐W basin. Sediment mercury levels also varied with several orders of magnitude, with the lowest mean concentration measured at the Voorste Neet (22.6 ± 24.2 µg/kg DS) and the highest at the Oude Schelde (747.9 ± 280.3 µg/kg DS) (Table S1). The contamination in the sediment was significantly different between basins (*F*_2,102_ = 14.5, *p *< 0.001), with a significantly higher degree of pollution in the Scheldt‐W basin (Tukey Honest Significant Difference test Scheldt‐W/Maas: *p* < 10−5; Scheldt‐W/Scheldt‐E: *p* = 0.007). The linear regression model with fish length as an explanatory variable, and the mercury content in the sediment used as covariate, explained almost 60% of the variation in muscle mercury concentration (adj. *R*² = 0.59, *F*_22,484 _= 35.23, *p* < 0.001). The length of the fish showed a significant positive correlation with the muscle mercury concentration at all locations, but one (osc) (Figure 3b). The mercury in the sediment did not significantly explain the variation in the muscle tissue (*p *= 0.13).

### 3.3 Genome‐wide association study

**FIGURE 4:**
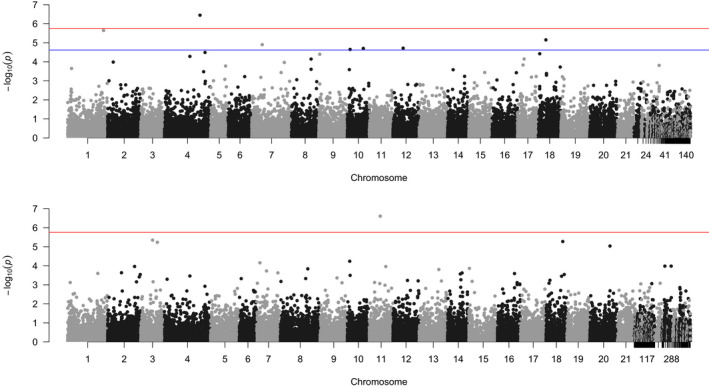
(a) Manhattan plot of the linear mixed model *p*‐values over their chromosomal position. The red line represents the –log10 of the Bonferroni corrected *p*‐value threshold (0.05/28450). (b) Manhattan plot of the latent factor mixed model *p*‐values over their chromosomal position. The plot convention exactly follows those of the plot in (a)

The GWAS results obtained by running the linear mixed model across all markers proved successful in correcting for *p*‐value inflation. A logQQ plot (Figure S4a) of the results indicates that our analysis effectively corrects for population structure and environmental confounders, because the overall distribution of the ‐log_10_ of our *p*‐values falls on the null distribution expectation. The logQQ plot provides a strong indication that the SNPs show a strong deviation from the null distribution and are associated with mercury in muscle. Therefore, to select the most likely candidates, we ran 10^4^ simulations of 28,450 samples (to match the number of our SNPs) sampled from a uniform distribution (which matches a purely random distribution of *p*‐values), and we assessed the distribution of the highest ‐log_10_ *p*‐value obtained from these simulations. Our distribution gave a median value of 4.621 and a 95^th^ percentile of 5.756; thus, SNPs producing a ‐log_10_ *p*‐value of association greater than 5.756 would be a true positive 95% or more of the times (any SNP with such significant association would also be significant after Bonferroni correction), and SNPs producing a ‐log_10_ *p*‐value of association greater than 4.621 would be a true positive 50% of the times. Based on these standards, we observe the top hit, on Chromosome 4, position (in bp) 25,127,344, has a ‐log_10_ *p*‐value of association of 6.45 (Figure S4a), exceeding the 95^th^ percentile of 5.756 (Figure S5). The effect size of this SNP on the variance of the z‐score transformed mercury content was 24%. We also observe that seven more SNPs are suggestive of association based on the 50% percentile threshold: SNPs groupI_25300153 (Chromosome 1, position 25,300,153, −log_10_ *p*‐value = 5.65), groupIV_28681469 (Chromosome 4, position 28,681,469, −log_10_ *p*‐value = 4.49), groupVII_7337531 (Chromosome 7, position 7,337,531, −log_10_ *p*‐value = 4.90), groupX_1922387 (Chromosome 10, position 1,922,387, −log_10_ *p*‐value = 4.65), groupX_11239053 (Chromosome 10, position 11,239,053, −log_10_ *p*‐value = 4.70), groupXII_7168446 (Chromosome 12, position 7,168,446, −log_10_ *p*‐value = 4.72) and groupXVIII_5496152 (Chromosome 18, position 5,496,152, −log_10_ *p*‐value = 5.15) (Table 1). Because these SNPs only reach a suggestive association we did not calculate their effect size. The LFMM analysis highlighted a SNP on Chromosome 11, with a ‐log_10_ *p*‐value of 6.61 (Figure 4b, Figure S4b, and Table 1). Despite the significant *p*‐value, this SNP has a very low effect size (0.1%), and it is dependent on nine minor allele homozygous individuals, all with a high mercury in muscle, and all found in the river Lede.

**TABLE 1:** Most significant hits from the mixed linear model analysis, the LFMM analysis, and the OutFLANK analysis of the SNPs of three‐spined stickleback. For each SNP we report the –log10(*p*), and any information about genes found within 5 kb from the SNP

Analysis	SNPID	*p*‐value	log_10_(*p*)	Gene region	GeneID Ensembl	Gene description	Gene location bp	Sequence ID	UniProt ID
Mixed linear model	groupI_25300153	2.24 × 10^−6^	5.65	NA	NA	NA	NA	NA	NA
	groupIV_25127344	3.55 × 10^−7^	6.45	NA	NA	NA	NA	NA	NA
	groupIV_28681469	3.24 × 10^−5^	4.49	Intron	wnk1b	WNK lysine‐deficient protein kinase 1b	Group IV: 28,658,720–28,716,045	ENSGACG00000019674	F1Q6T6
	groupVII_7337531	1.26 × 10^−5^	4.90	Intron	pcxb	pyruvate carboxylase b	Group VII: 7,239,835–7,431,018	ENSGACG00000019710	F1QYZ6
	groupX_1922387	2.24 × 10^−5^	4.65	Down stream	si:dkeyp−92c9.2	Cyclin‐dependent kinase 5 activator	Group X: 1,920,938–1,921,900	ENSGACG00000002355	Q1LWT0
	groupX_11239053	1.99 × 10^−5^	4.70	exon	dlgap3	discs, large (Drosophila) homolog‐associated protein 3	Group X: 11,228,435–11,239,167	ENSGACG00000007439	O95886
	groupXII_7168446	1.91 × 10^−5^	4.72	intron	amt	Aminomethyl transferase	Group XII: 7,166,152–7,171,302	ENSGACG00000005808	P48728
	groupXVIII_5496152	7.08 × 10^−6^	5.15	Down stream	fam89a	family with sequence similarity 89 member A	Group XVIII: 5,493,939–5,496,151	ENSGACG00000006868	Q14BJ1
LFMM	groupXI_7668256	2.45 × 10^−7^	6.61	NA	NA	NA	NA	NA	NA
OutFLANK	groupIV_31526711	5.24 × 10^−7^	6.28	exon	kdm7ab	lysine (K)‐specific demethylase 7Ab	Group IV: 31,515,284–31,530,816	ENSGACG00000019975	F1QHF6

### 3.4 *F*_st_ outlier test

Finally, the outlier test identified one SNP that appeared under selection when the samples were divided by the 21 sampling locations. This single SNP does not match those found by the mixed model approaches, but we still identified a signal from Chromosome 4 (on position 31,526,711, ‐log_10_ *p*‐value of 6.58) (Table 1). On the other hand, when samples were divided according to the three drainage basins we observed that no any SNP was significantly under selection.

### 3.5 Gene ontology analysis

An in‐depth investigation of the locations of the best hits showed that seven out of ten SNPs were positioned within a 2500 bp distance of an annotated gene (Table 1). They were found both on noncoding intron regions, mRNA coding (exon) regions and 1 bp behind a known gene sequence (groupXVIII_5496152). The two mutations located at exon regions, groupX_11239053 and groupIV_3152526711, were identified as silent mutations. Finally, the region on the outer end of Chromosome 4 (between 25,127,344 and 31,526,711 bp) is putatively the most important with respect to mercury accumulation in the muscle tissue and was therefore screened in detail for genes related to internal metal handling. In total, 290 genes were found on Chromosome 4, which are part of a wide variety of biological and cellular processes. Yet, only 13 (Table 2) are assumed to code for proteins interacting selectively and noncovalently with metal ions. Two GWAS SNPs (groupIV_28915071, groupIV_28952905) located within the boundaries of a gene coding for cacna1c were significantly associated with mercury accumulation in the muscle tissue, but the associated *p*‐values were only of minor significance.

**TABLE 2:** List of annotated genes (Ensembl) that were located within the Chromosome 4 region that showed a strong association with mercury in the muscle tissue (25,127,344 and 31,526,711 bp)

Gene name	GO term name	GO term definition	SNP_ID and log10*(p*) of GWAS SNPs	Gene start (bp)	Gene end (bp)
cmah	Metal ion binding	Interacting selectively and noncovalently with any metal ion.	NA	25,436,299	25,450,435
osgep	Metal ion binding	Interacting selectively and noncovalently with any metal ion.	NA	25,499,574	25,509,648
apex1	Metal ion binding	Interacting selectively and noncovalently with any metal ion.	NA	25,509,908	25,512,909
rnf103	Metal ion binding / Zinc ion binding	Interacting selectively and noncovalently with any metal ion. / Interacting selectively and noncovalently with zinc (Zn) ions.	NA	26,185,449	26,190,872
ENSGACG00000019612	Zinc ion binding	Interacting selectively and noncovalently with zinc (Zn) ions.	NA	26,816,361	26,817,952
napepld	Zinc ion binding	Interacting selectively and noncovalently with zinc (Zn) ions.	NA	28,736,626	28,741,815
cacna1c	Metal ion binding	Interacting selectively and noncovalently with any metal ion.	**groupIV_28915071 (1.9), groupIV_28952905 (1.6)**, groupIV_28952926 (0.02)	28,882,245	28,979,573
zc3hc1	Metal ion binding / Zinc ion binding	Interacting selectively and noncovalently with any metal ion. / Interacting selectively and noncovalently with zinc (Zn) ions.	groupIV_29035551 (0.7), groupIV_29035570 (0.7), groupIV_29035587 (0.7), groupIV_29035587 (0.7)	29,033,967	29,037,601
ENSGACG00000019741	Metal ion binding / Zinc ion binding	Interacting selectively and noncovalently with any metal ion. / Interacting selectively and noncovalently with zinc (Zn) ions.	NA	29,574,727	29,594,210
			NA		
pcloa	Metal ion binding	Interacting selectively and noncovalently with any metal ion.	NA	29,972,662	30,015,811
impdh1b	Metal ion binding	Interacting selectively and noncovalently with any metal ion.	groupIV_30417642 (0.05)	30,411,949	30,419,505
si:ch211‐244b2.4	Metal ion binding	Interacting selectively and noncovalently with any metal ion.	NA	30,593,947	30,596,648
ENSGACG00000019823	Zinc ion binding	Interacting selectively and noncovalently with zinc (Zn) ions.	groupIV_30599051 (0.09), groupIV_30599052 (0.2)	30,597,678	30,600,528
ENSGACG00000019938	Metal ion binding / Zinc ion binding	Interacting selectively and noncovalently with any metal ion. / Interacting selectively and noncovalently with zinc (Zn) ions.	groupIV_31122917 (0.4), groupIV_31122983 (0.9), groupIV_31123011 (0.7)	31,122,014	31,125,008
ENSGACG00000019975	Metal ion binding	Interacting selectively and noncovalently with any metal ion.	groupIV_31526591 (1.0), groupIV_31526633 (1.3), groupIV_31526639 (1.0), groupIV_31526711 (0.05)	31,515,284	31,530,816

Note

Gene ontology (GO) term name and definition were enquired with Biomart from the Ensembl database. The SNPs located within the gene's start and end positions were extracted from our SNP dataset and loci significantly (*p* < 0.05) associated with mercury in muscle tissue are highlighted in bold.

## 4 DISCUSSION

Our analysis confirmed our first hypothesis that we would be able to find loci associated with the accumulation of mercury in muscle. It did not provide strong support for our second hypothesis, that adaptation to mercury pollution would cause a different pattern of association in the three main river basins of Flanders.

### 4.1 Mercury pollution

We monitored a highly diverse set of habitats in terms of environmental pollution with a wide range of mercury levels measured in the sediment (22.6–748 µg/kg DS). However, only at one location (osc) the Flemish sediment quality guideline (550 µg/kg) was consistently exceeded in the majority of the sediment samples and thus indicates a potential risk to the environment (De Deckere et al., 2011). The mercury levels in the fish muscle tissue also showed a great variability across locations, with an order of magnitude of difference between locations (abe: 22.04 ng/g to mlb: 326.1 ng/g DW) (Table S1). The two locations where muscle mercury levels were the highest, Lede (Scheldt‐W) and Molenbeek (Scheldt‐E), showed bioaccumulation profiles of the same range as measured in three‐spined sticklebacks of polluted waters elsewhere (Kenney et al., 2012, 2014; Willacker et al., 2013). These were lower levels than those measured in European eel (*Anguilla anguilla*) and perch (*Perca fluviatilis*) of Belgian surface waters (Teunen et al., 2017). While we expected to find high levels of mercury in the river Lede, a legacy of the felt industry at the city of Lokeren (Van Dyck, 2012), no obvious source of mercury pollution could be identified for the Molenbeek. Although mercury accumulation in the fish from the rivers Lede and Molenbeek was considerably elevated, the mean tissue concentrations of 17 out of 21 locations exceeded the biota quality standard (BQS) of 20 ng/g wet weight (EU: Directive 2000/60/ec). Since this BQS was included in the Water Framework Directive to protect predatory fish, birds and humans from secondary poisoning, these mercury levels in the fish suggest a higher ecological risk than we expect based on environmental levels alone. The discrepancy between mercury levels in the environment (sediment) and biota is statistically illustrated by our regression model that shows no significant effect of mercury content in the sediment on bio‐accumulated mercury. This result is not entirely surprising and is mainly attributable to the complex geochemistry of mercury in aquatic environments. Water variables such as pH and oxygen levels along with sediment variables such as sulphide levels and organic matter all have an effect on the fractionation of mercury species and their bioavailability. These specific environmental conditions will govern the amount of inorganic mercury that is available for uptake by methylating bacteria who convert it to the organic methylmercury. The latter is the predominant mercury form that biomagnifies through the food web (Chiasson‐Gould et al., 2014; Dranguet et al., 2017; Hsu‐Kim et al., 2013). Furthermore, specific food web characteristics such as biodilution at the base of the food chain, and habitat specific macroinvertebrate composition are also assumed to explain differences in mercury transfer through the aquatic food web (Chouvelon et al., 2018; Jedruch et al., 2019). The importance of dietary uptake is clearly evidenced in our regression model by the significant effect of fish length. Previous research showed that mercury, and methylmercury in particular, is only slowly eliminated from the body (Garnero et al., 2020; Trudel & Rasmussen, 1997). Therefore, growing fish bioaccumulate mercury in their tissue and as such levels progressively increase with age. Although exact age determination was not possible, fish length is directly correlated with age (Isermann & Knight, 2005; Ogle, 2018). Our findings, that mercury content in muscles is strongly associated with length, provide solid evidence that length is a good proxy for age and is an effective measure to correct for exposure time to dietary mercury.

### 4.2 Genomics of adaptation to mercury

We know that various fish species thrive in highly polluted waters, despite the exposure to cocktails of pollutants, in some cases for centuries, and in cases where polluted waters would elicit a toxic response from naive animals (Durrant et al., 2011; Wirgin & Waldman, 2004). For instance, evidence is mounting that some populations of the Atlantic killifish (*Fundulus heteroclitus*) have evolved pollution tolerance (Reid et al., 2017; Whitehead et al., 2012), a result suggesting that pollutants are acting as a selective agent on natural populations, and that at least some natural populations are adapting to this selective challenge. Thus, there is empirical evidence that pollutants may act as one of the selective forces in the environment and that genetic adaptation to pollutants is both possible and can occur naturally. Empirical results from family based experiments also show that accumulation of mercury in the body is highly heritable in steelheads (Blanc et al., 2003) and zebra finches (Buck et al., 2016). The fact that a strong genetic component is observed in such disparate taxa strengthens the assumption that genetic differences generally affect mercury accumulations in vertebrates. In these studies the authors suggested that fitness advantage associated with this trait was related to an increased tolerance to mercury accumulation. However, due to the lack of identification of a responsible gene or pathway, this explanation still remains speculative. Our first hypothesis therefore tested whether we identify an association between genotype and mercury accumulation in muscle. It was supported by our data; we identified that a region of Chromosome 4 is consistently associated with the amount of mercury fish accumulated in muscle tissue. Two SNPs were identified with two different approaches (the linear mixed model and the outlier analysis), both giving *p*‐values exceeding genome‐wide significance thresholds. It is unlikely that the SNPs we identified are directly causal, or closely linked with the locus, or loci, that modulate the accumulation of mercury in muscle. Nevertheless, our results show such strong and consistent association on Chromosome 4, that it is extremely likely that the locus, or loci, controlling this phenotype, are located on this chromosome, putatively between 25,127,344 and 31,526,711 bp. Additionally, these two SNPs explain, respectively, 24% and 21% of the normalized variance in mercury accumulation in muscles, suggesting that the actual causal locus, or loci, can have an important role in mercury accumulation in muscle. We highlighted a number of loci associated with metal metabolism (Table 2), but none of the SNPs falling near these genes show any evidence of association with mercury in muscle. On the other hand, a published QTL on the right arm of Chromosome 4 is associated with the number and spacing of pharyngeal teeth (Cleves et al., 2014). Food is known to be the main path of exposure to methylmercury in fish (Bradley et al., 2017; Keva et al., 2017; Kozak et al., 2021). Our data show that mercury in muscle is proportionally higher in larger fish, a result that indicates that mercury is accumulated as the fish grows, and is consistent with food as the main source of mercury. The diet of the fish and exact methylmercury load in the food available in our sampling locations have not been characterized. It is possible that the pharyngeal teeth morphology modulates the uptake of mercury in the diet and that our results highlight this association. Yet, we cannot assess whether mercury accumulation in muscle should be considered an adaptive response in itself, or whether it is the by‐product of other biological functions (potentially the pharyngeal dentition), which are under mercury selection, or whether it simply indicates that some fish can tolerate a higher load of mercury. Our second hypothesis, that of parallel but independent genetic mechanisms controlling mercury accumulation, was the result of the observation that parallel adaptations occur in nature (Alves et al., 2019; Chan et al., 2010; Zhang et al., 2021), even for adaptation to pollution (Reid et al., 2017). Nevertheless, the support provided to the data by this hypothesis is weaker and cannot unequivocally conclude that we see parallel genetic adaptation mechanisms. The results from the linear mixed model suggest that the genetic mechanism of mercury in muscle accumulation is either the same or it is at least controlled by different loci all located on Chromosome 4 across all our sampling sites. The reason is that the locus on Chromosome 4, position 25,127,344 shows variability across all sampling sites. The results from the outlier test performed on the samples divided by drainage basin also found no markers showing a signal of selection. We cannot exclude that there are potentially other loci acting on mercury in muscle accumulation at the population level. The outlier test performed on the samples by sampling location identified one locus on Chromosome 4, position 31,526,711, which is fixed for all populations apart from the Abeek and Itterbeek samples, both part of the Maas drainage. Because this marker was not identified by the basin‐clustered analysis, its selective value might not be strong enough to cause a significant effect on the *F*_st_ when clustering is basin based. A second indication of ‘private’ alleles comes from the results of the LFMM analysis. Nevertheless, the only locus found by LFMM analysis explains a trivially small percentage of the mercury variance; it seems to act in a recessive fashion. Overall, we interpret these results as being broadly against the idea of parallel adaptation. However, we cannot exclude the possibility that, at the local level, other genetic mechanisms are involved in mercury accumulation in muscle. Overall, our results provide the basis for further common garden experiments to assess causal relationships between alleles and mercury accumulation, and help identify the actual mechanisms underlying these causal relationships.

### 4.3 Mercury pollution, fish and management

Our results strongly suggest that monitoring mercury in the environment is best carried out by using a sentinel species such as the three‐spined stickleback rather than by simply measuring mercury pollution in the sediment. It is not understood why we do not observe a direct correlation between mercury in the sediment and mercury in muscle. However, it is known that methylation plays a key role in the mobilization of this pollutant in the food chain (Hoffman et al., 2002). The availability of methylated mercury might be more important than the total mercury present in the environment. Because the most important effect of mercury as a pollutant is its biomagnification through the food chain, extending the use of three‐spined sticklebacks as monitor species for this metal would provide a more effective approach, especially in terms of environmental monitoring (Sanchez et al., 2008) and public health (Morrens et al., 2012). A second important consideration, which will almost surely need to be explored with a wider, multi‐species approach, is that adaptation to mercury does happen. Therefore, the presence or absence of a reasonable proportion of the original riverine biodiversity cannot be taken by itself as an indicator of good environmental conditions, especially in a region like Flanders, with pollution that is both historical and pervasive (Sonke et al., 2003).

## 5 CONCLUSION

The finding of a locus on Chromosome 4 and possibly Chromosome 11 linked to mercury accumulation in muscle tissue of fish provides the rationale for further studies based on targeted resequencing. Such approach will narrow the genomic area associated with mercury accumulation and potentially identify genes whose causal effects can be tested by mercury challenge tests. The possibility that diet (pharyngeal teeth) is involved also opens up the opportunity to directly assess the association between this phenotype and mercury accumulation, and, once again, to test for a direct causality experimentally. A firm proof of adaptation will require a strategy involving a higher resolved genetic characterization through resequencing, biomarker assessments and common garden experiments with adapted and control fish. Finally, and irrespective of the actual causal variants and the actual mechanism controlling mercury accumulation in muscle, it will also be important to understand the potential costs of and constraints to adaptation to mercury, to fully understand how and why specific loci and alleles have been selected in response to mercury, and to be able eventually to place these results in an eco‐evolutionary framework.

## CONFLICT OF INTEREST

The authors have declared no conflict of interest.

## Supporting information

Supplementary Material

## Data Availability

The data that support the findings of this study are openly available in Dryad at https://doi.org/10.5061/dryad.6t1g1jx06
