# Supplementary material for: Association between Chromosome 4 and mercury accumulation in muscle of the three‐spined stickleback (Gasterosteus aculeatus)
Source: Evol Appl. 2021 Oct 9;14(10):2553–67. doi: 10.1111/eva.13298 (PMC8549617; doi:10.1111/eva.13298)
Supplement: Supplementary file 1 — Supplementary Material [file EVA-14-2553-s001.pdf]

Supplementary Information

Figure S1: Manhattan plot of association between genotype and phenotypic sex. As expected, SNPs on Chromosome 19 are strongly associated with phenotypic sex.

Figure S2: PCA plot (PC1 and PC2) of the population structure of all samples, inclusive of the data on Chromosome 19. Populations represented with triangles of a shade of blue have been sampled in the Scheldt-W basin; populations represented with diamonds of a shade of green have been sampled in the Scheldt-E basin; populations represented by circles of a shade of brown have been sampled in the Maas basin.

Figure S3 A: PCA plot (PC3 and PC5) of the population structure of all samples, inclusive of the data on Chromosome 19. The green line visually divides the males from the females as called by gonad phenotype. **Figure S3 B**: Same plot with the samples divided by sex – it is possible to see how this method of sexing the fish is not always correct, due to the fact the fish might have been too small or immature at the time of dissection.

Figure S4: **A** LogQQ plot of the p-values obtained by the linear mixed model used to assess association between genotype and mercury in muscle. On the X axis are the expected values, on the Y axes the observed values (both as $-\log_{10}$(p-value)). The deviation from the null expectation (the red line) on the right hand of the plot indicates that the p-values of these tests are more significant than what we would expect by chance alone. **B** LogQQ plot of the p-values obtained by latent factor mixed model to assess association between genotype and mercury in muscle. The plot convention exactly follows those of the plot in A.

Figure S5: Distribution of the smallest $-\log_{10}$(p-value) of 28450 p-values randomly drawn from a uniform distribution, repeated 10,000 times. The black line represents the 95_{th} percentile, the red line represents the p-value actually observed in the most significant SNP identified by the mixed linear model.

Table S1: Environmental parameters measured in the water and sediment at the 21 sampling sites of three-spined stickleback. Information includes the basin, site name, site unique identifier, coordinates, mercury in sediment, pH and temperature at the time of sampling,

Table S2: Information on the recoveries obtained from the standard reference material measured for quality control purpose. Certified freeze-dried mussel tissue (BCR 2976, NIST, USA) and channel sediment (BCR-320R, IRMM, Geel Belgium) were used for the muscle tissue and sediment samples respectively.

Table S3: Pairwise F_{st} between populations.

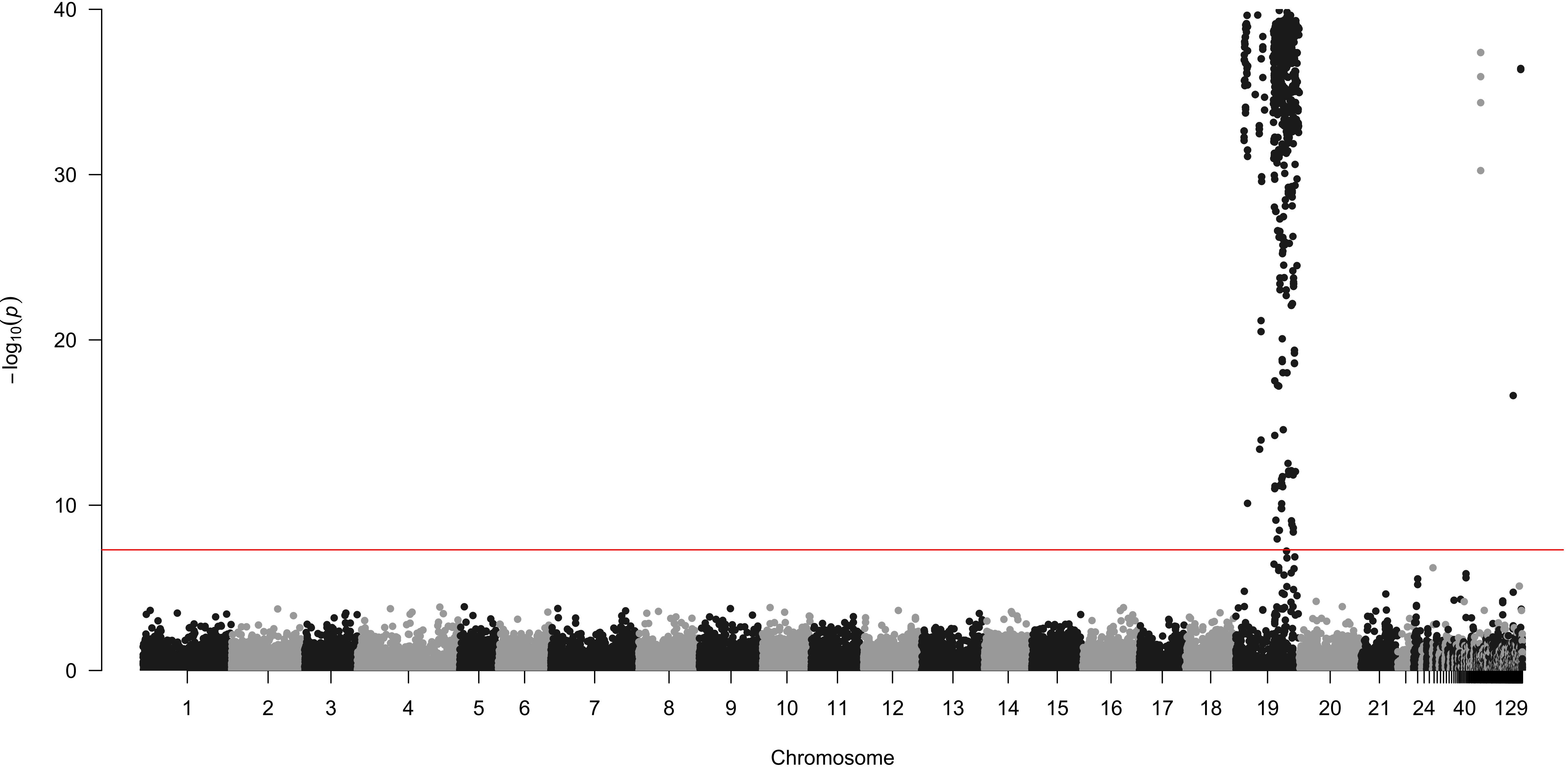

40
30
20
10
0
$-\log_{10}(p)$
1
2
3
4
5
6
7
8
9
10
11
12
13
14
15
16
17
18
19
20
21
24
40
129
Chromosome

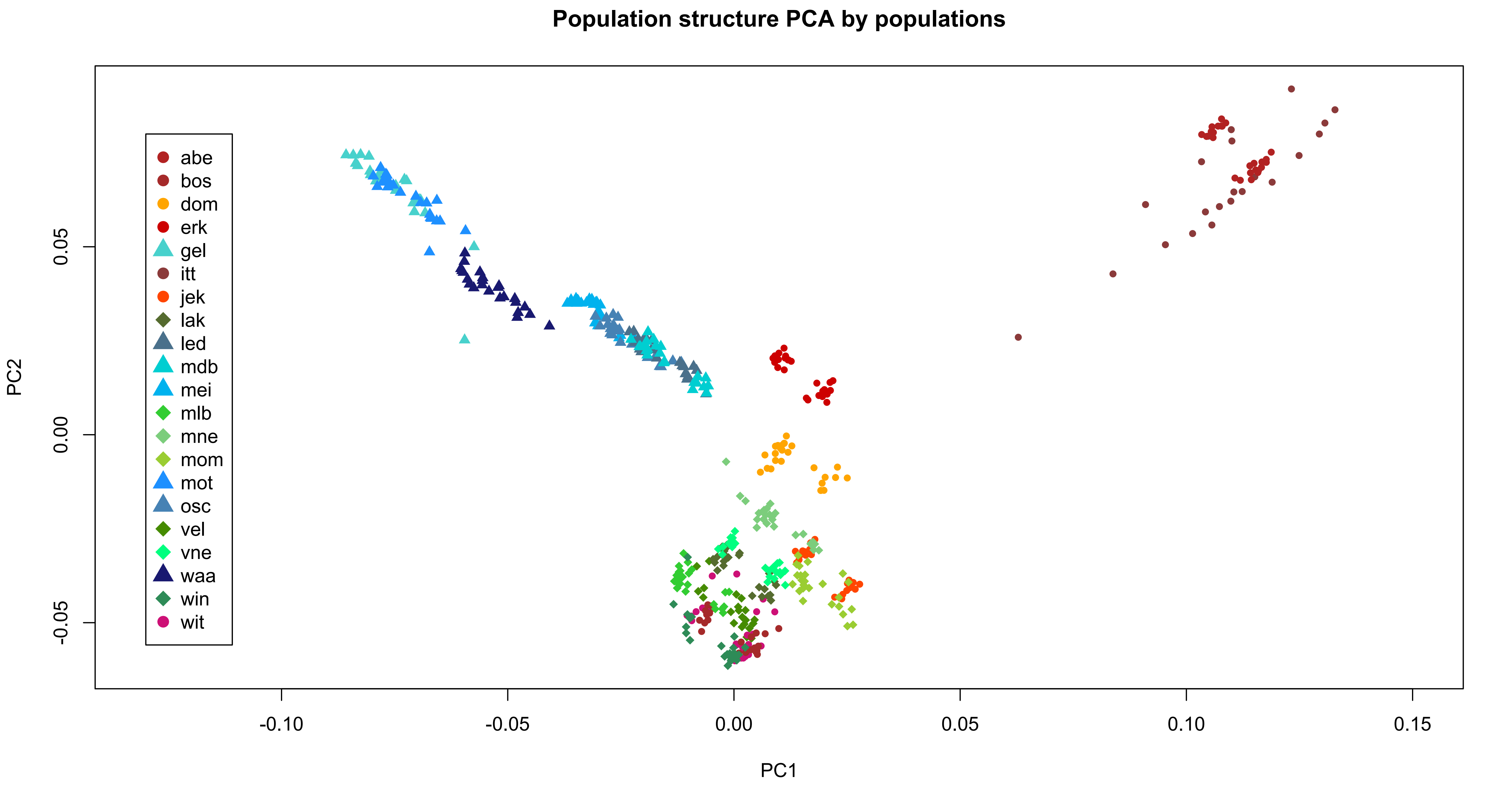
Population structure PCA by populations
abe
bos
dom
erk
gel
itt
jek
lak
led
mdb
mei
mlb
mne
mom
mot
osc
vel
vne
waa
win
wit
0.05
0.00
-0.05
PC2
-0.10
-0.05
0.00
0.05
0.10
0.15
PC1

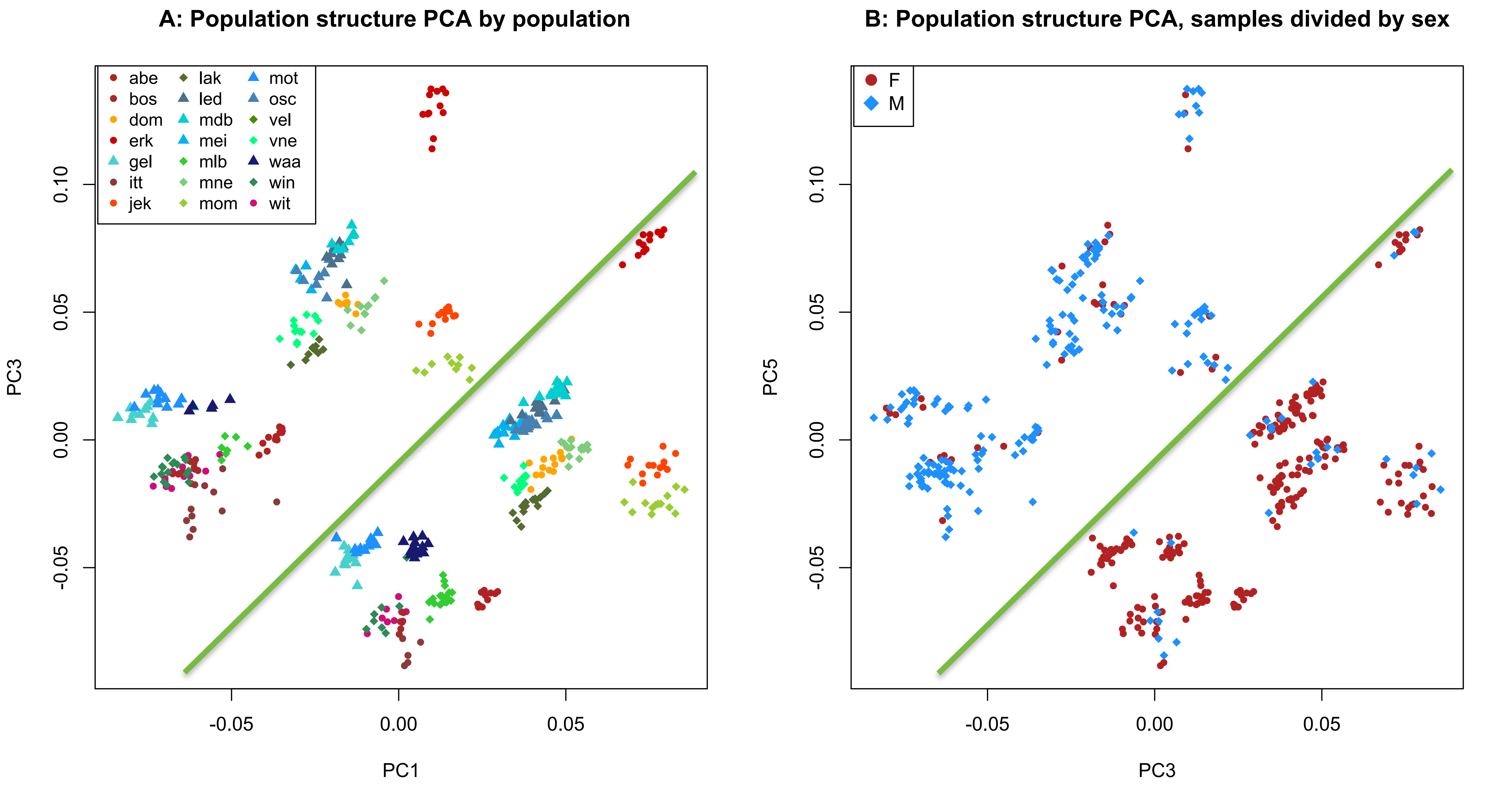

A: Population structure PCA by population
abe
bos
dom
erk
gel
itt
jek
lak
led
mdb
mei
mlb
mne
mom
mot
osc
vel
vne
waa
win
wit
PC1
PC3
-0.05
0.00
0.05
0.10
B: Population structure PCA, samples divided by sex
F
M
PC3
PC5

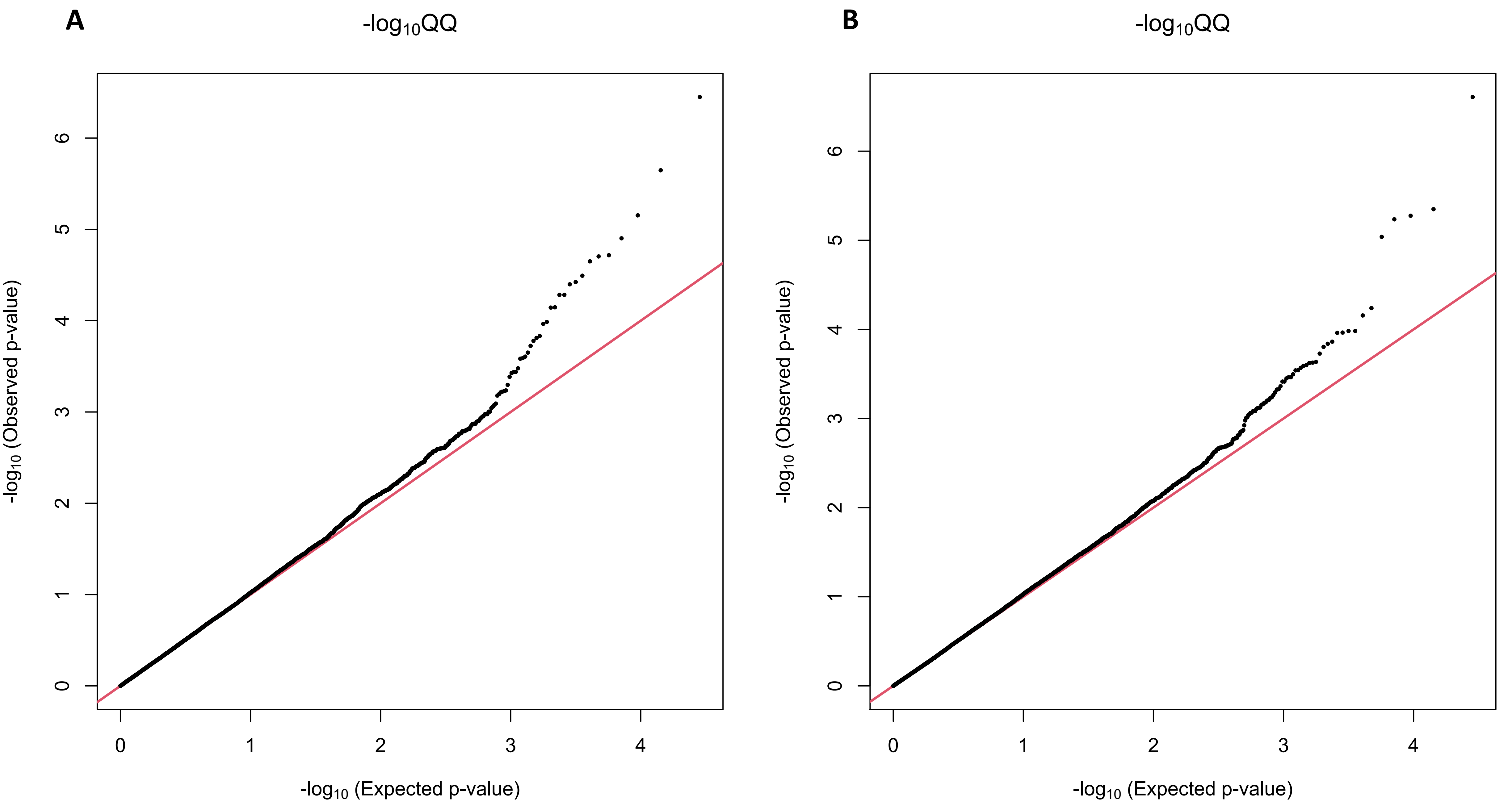
A
-log$_{10}$QQ
-log$_{10}$ (Observed p-value)
-log$_{10}$ (Expected p-value)
0
1
2
3
4
5
6
B
-log$_{10}$QQ
-log$_{10}$ (Observed p-value)
-log$_{10}$ (Expected p-value)
0
1
2
3
4
5
6

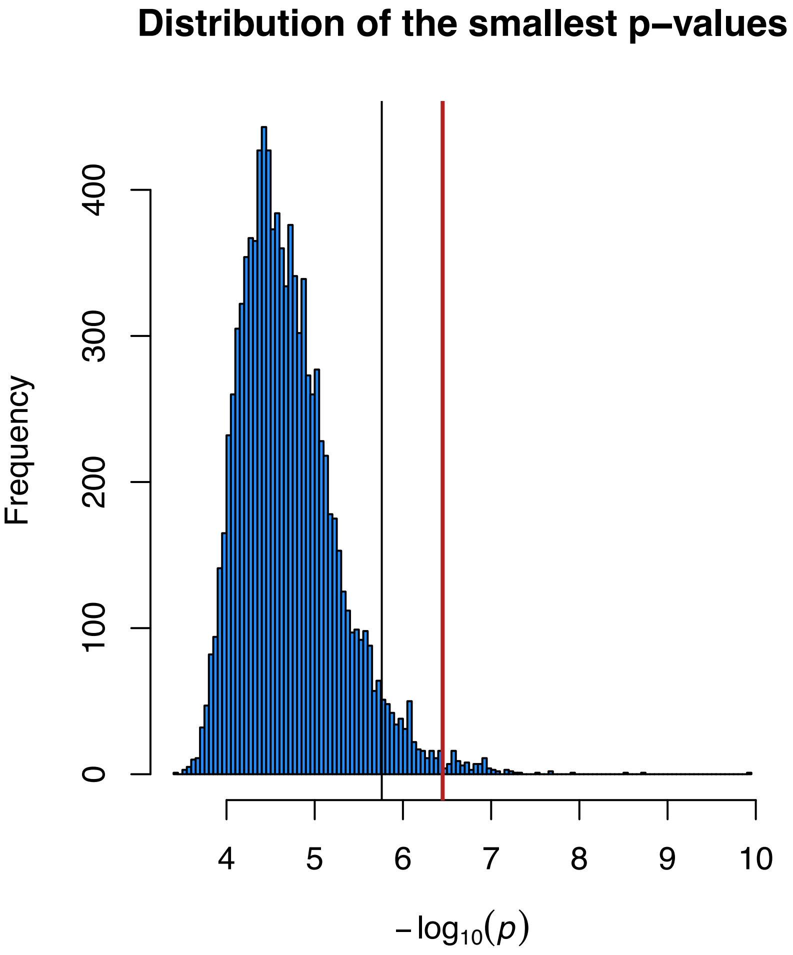

Distribution of the smallest p−values
Frequency
0
100
200
300
400
4
5
6
7
8
9
10
$-\log_{10}(p)$
